# Identification and Characterization of NTB451 as a Potential Inhibitor of Necroptosis

**DOI:** 10.3390/molecules23112884

**Published:** 2018-11-05

**Authors:** Eun-Jung In, Yuno Lee, Sushruta Koppula, Tae-Yeon Kim, Jun-Hyuk Han, Kwang-Ho Lee, Tae-Bong Kang

**Affiliations:** 1 BK21PLUS Glocal Education Program of Nutraceuticals Development, Department of Applied Life Science, Graduate School, Konkuk University, Chungju 27478, Korea; dlsdmswjd12@gmail.com (E.-J.I.); xodus1376@naver.com (T.-Y.K.); digit0516@naver.com (J.-H.H.); 2 Korea Chemical Bank, Korea Research Institute of Chemical Technology, Daejeon 34114, Korea; yunolee1@krict.re.kr; 3 Department of Biotechnology, College of Biomedical & Health Science, Research Institute of Inflammatory Diseases, 268 Chungwon-daero, Chungju 27478, Korea; sushrutak@gmail.com

**Keywords:** necroptosis, inhibitor, NTB451, RIPK1

## Abstract

Necroptosis, or caspase-independent programmed cell death, is known to be involved in various pathological conditions, such as ischemia/reperfusion injury, myocardial infarction, atherosclerosis, and inflammatory bowel diseases. Although several inhibitors of necroptosis have been identified, none of them are currently in clinical use. In the present study, we identified a new compound, 4-({[5-(4-aminophenyl)-4-ethyl-4H-1,2,4-triazol-3-yl]sulfanyl}methyl)-*N*-(1,3-thiazol-2-yl) benzamide (NTB451), with significant inhibitory activity on the necroptosis induced by various triggers, such as tumor necrosis factor-α (TNF-α) and toll-like receptor (TLR) agonists. Mechanistic studies revealed that NTB451 inhibited phosphorylation and oligomerization of mixed lineage kinase domain like (MLKL), and this activity was linked to its inhibitory effect on the formation of the receptor interacting serine/threonine-protein kinase 1 (RIPK1)-RIPK3 complex. Small interfering RNA (siRNA)-mediated RIPK1 knockdown, drug affinity responsive target stability assay, and molecular dynamics (MD) simulation study illustrated that RIPK1 is a specific target of NTB451. Moreover, MD simulation showed a direct interaction of NTB451 and RIPK1. Further experiments to ensure that the inhibitory effect of NTB451 was restricted to necroptosis and NTB451 had no effect on nuclear factor-κB (NF-κB) activation or apoptotic cell death upon triggering with TNF-α were also performed. Considering the data obtained, our study confirmed the potential of NTB451 as a new necroptosis inhibitor, suggesting its therapeutic implications for pathological conditions induced by necroptotic cell death.

## 1. Introduction

Programmed cell death is an important biological process in tissue homeostasis, animal development, and disease [1,2,3]. Necroptosis, one of the programmed necrosis types, is characterized by membrane and organelle swelling, leading to membrane rupture and the release of intracellular contents called damage-associated molecular patterns (DAMPs). Receptor-interacting serine/threonine-protein kinase 1 (RIPK1), RIPK3, and mixed lineage kinase domain-like (MLKL) are known to be the key molecules in the necroptosis signaling pathway [4,5]. Many stimuli, such as tumor necrosis factor (TNF), Fas ligand (FasL), TNF-related apoptosis-inducing ligand (TRAIL), interferon (IFN), double-strand RNA (dsRNA) and lipopolysaccharide (LPS) can trigger necroptosis in cells [6]. Among these, TNF-induced necroptosis is a well-studied pathway. The ligation of TNF receptor 1 (TNFR1) by TNF promotes the recruitment of TNFR1-associated death domain protein (TRADD), TNF receptor -associated factor 2 (TRAF2), RIPK1, and cellular inhibitor of apoptosis 1 and 2 (cIAP1/2) to form receptor complex I [7]. The internalization of complex I following cleavage of ubiquitin from RIPK1 by deubiquitinating enzymes, such as A20 or CYLD, leads to the formation of complex II, which is composed of RIPK1, caspase-8, and Fas-associated protein with death domain (FADD) [8,9,10,11]. When caspase activity is inhibited, RIPK1 and RIPK3 are recruited and activated by phosphorylation; the activated RIPK3 then phosphorylates MLKL [4,5,12,13]. Phosphorylated MLKL is oligomerized and translocated to the plasma membrane, which is an essential event in the induction of necroptosis [14,15].

Necroptosis has been related to diverse pathological conditions, such as ischemia/reperfusion kidney injury, stroke, atherosclerosis, Huntington’s disease, colitis, steatohepatitis, and acute hepatitis [16,17,18,19,20,21,22]. Therefore, the study of necroptosis inhibitors is important for clinical drug development. Several inhibitors suppress necroptosis in vitro and in vivo. For example, studies have reported that necrostatin-1 (Nec-1) and its analogs inhibit necroptosis by targeting RIPK1 in vitro [23]. Research also reported that Nec-1 ameliorated renal and brain ischemia/reperfusion injury [17,24], ConA-induced hepatitis [22], DSS-induced colitis [20], and cell death following retinal detachment in rats [25]. Further, Nec-1 decreased the symptoms of Huntington’s disease in a murine study, resulting in the maintenance of body weight [19].

The RIPK3 inhibitor, N-(6-propan-2-ylsulfonylquinolin-4-yl)-1,3-benzothiazol-5-amine (GSK’872), attenuates ischemic brain injury by downregulating HIF-1α and necroptosis-related proteins [26]. Dabrafenib conferred protection against acetaminophen-induced liver injury by targeting RIPK3 [27]. Necrosulfonamide blocked necroptosis in human cells via the inhibition of MLKL activation [5]. In addition, two Food and Drug Administration (FDA)-approved drugs, ponatinib and pazopanib, specifically inhibited necroptosis by targeting RIP1 and RIP3 kinase activity in vitro [28]. However, none of these inhibitors entered into the clinical setup. In the present study, we identify a new compound, 4-({[5-(4-aminophenyl)-4-ethyl-4H-1,2,4-triazol-3-yl] sulfanyl}methyl)-N-(1,3-thiazol-2-yl) benzamide (NTB451), as a potential necroptosis inhibitor and reveal the mechanism of action in several aspects of cellular models.

## 2. Results

### 2.1. NTB451 Inhibits TNF-Induced Necroptosis

**Figure 1:**
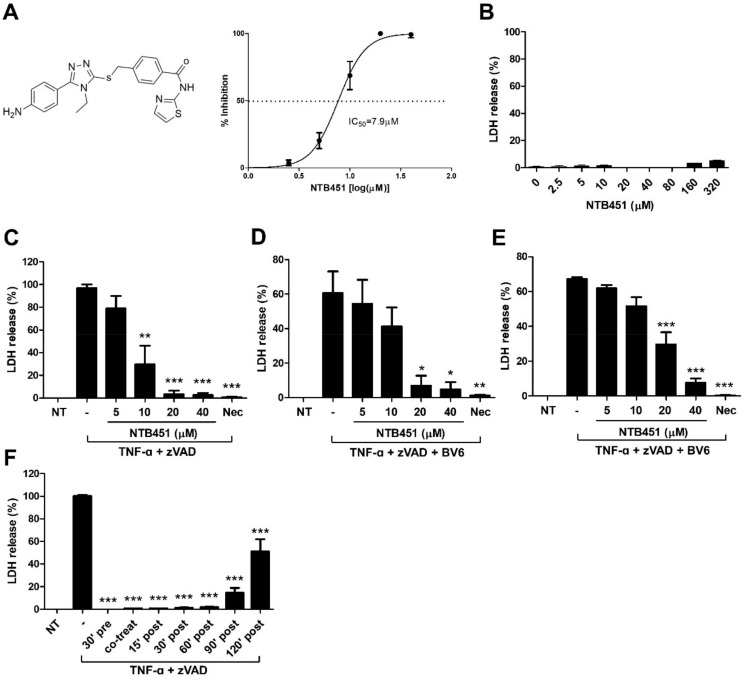
Identification of NTB451 as an inhibitor of TNF-induced necroptotic cell death and its effect on various cell lines. (**A**) Chemical structure of NTB451 and IC_50_ value. (**B**) Cell cytotoxicity of L929 cells by the treatment of NTB451 for 24 h. (**C**–**F**) The effect of NTB451 on necroptosis. (**C**) L929 cells were treated with TNF-α (400 units/mL) and zVAD (20 µM) for 5 h in the presence or absence of the indicated concentration of NTB451 or Nec-1 (10 µM). MEF (**D**) and HT-29 (**E**) cells were pre-treated with BV6 (1 µM) for 1 h and then exposed to TNF-α plus zVAD for 4 h and 15 h, respectively. (**F**) L929 cells were treated with TNF-α plus zVAD for 5 h. NTB451 (20 µM) was applied to the cells before and after treatment of TNF-α plus zVAD at the indicated timepoints. Cell death was then measured by lactate dehydrogenase (LDH) release in culture supernatants. The data are represented as the mean ± standard error of the mean (SEM) of two independent experiments performed in triplicate. * *p* < 0.05, ** *p* < 0.01, *** *p* < 0.001 compared with the group treated with TNF-α + zVAD or TNF-α + zVAD + BV6.

As described in the Materials and Methods Section, using L929 cells, the first-round necroptosis inhibitor screening was performed with chemical library comprising 8363 compounds and four compounds that showed higher than 50% inhibition of necroptotic cell death at a concentration of 50 µM were initially selected as hits. We therefore performed the second-round assay for dose-dependency and cytotoxicity with selected compounds. A promising compound that showed more than 80% inhibition of necroptosis with IC_50_ values less than 10 µM was identified, which was referred to as NTB451 (Figure 1A). NTB451 dose-dependently blocked the death of L929 cells induced with TNF-α and zVAD at a concentration that did not show any significant cytotoxic effect (Figure 1B,C). To investigate whether NTB451 had an inhibitory effect on cell death in cells other than L929, mouse embryonic fibroblast (MEF) cells and a human cell line (HT-29) that is often used in necroptosis studies were treated with a combination of TNF-α, zVAD, and Smac mimetic BV6 plus Nec-1, RIPK1 kinase inhibitor, or the indicated concentrations of NTB451. As expected, NTB451 significantly decreased TNF-induced necroptosis in both cell types in a dose-dependent manner (Figure 1D,E). To determine whether NTB451 had therapeutic properties, the cells were treated with NTB451 before or after TNF-α and zVAD stimulation. The inhibitory activity of NTB451 on the necroptosis was evaluated 2 h post-treatment of TNF-α (Figure 1F).

### 2.2. NTB451 Prevents TNF-α-Mediated Necroptosome Formation, Resulting in Inhibition of MLKL Phosphorylation and Oligomerization

**Figure 2:**
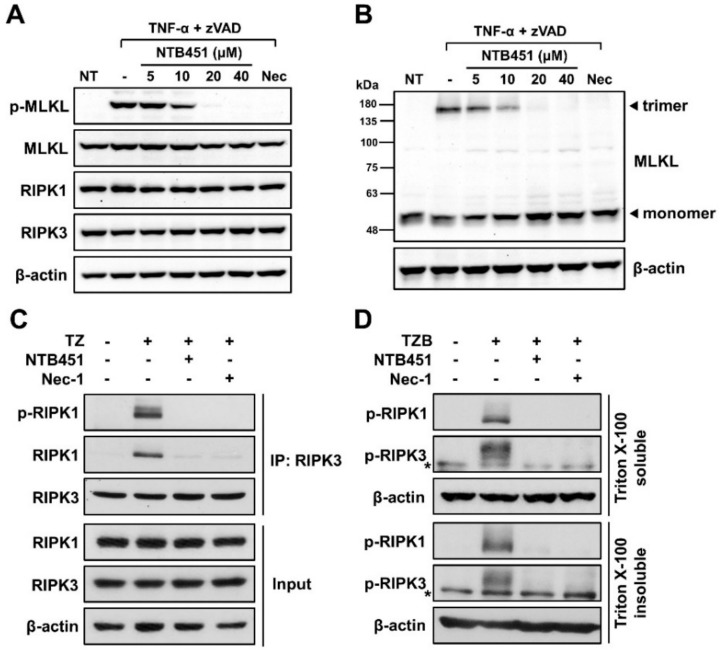
Effect of NTB451 on TNF-induced MLKL activation and the formation of necroptosome. (**A**–**C**) L929 cells were treated with TNF-α (400 units/mL) and zVAD (20 µM) for 2 h in the presence or absence of the indicated amounts of NTB451 or Nec-1 (10 µM), and cell lysates were prepared as described in the Materials and Methods Section 4.6. (**A**) Immunoblot analysis of phospho-MLKL, MLKL, RIPK1, or RIPK3. (**B**) Immunoblot analysis of MLKL under non-reducing conditions. (**C**) Necroptosome was immunoprecipitated with anti-RIPK3 antibody and probed with anti-phospho-RIPK1 or RIPK1 antibodies. (**D**) HT-29 cells were pretreated with BV6 (1 µM) for 1 h and then exposed with hTNF-α plus zVAD for 4 h 30 min in the presence or absence of NTB451 (40 µM) or Nec-1 (10 µM). Immunoblot analysis of phospho-RIPK1 or RIPK3 in Triton X-100 soluble and insoluble fractions. The soluble fractions were obtained by lysing cells with TTNE lysis buffer, and insoluble fractions were prepared by lysing insoluble pellets with 1% sodium dodecyl sulfate (SDS) lysis buffer. * indicates a nonspecific band.

Mounting evidence revealed that the two kinases, RIPK1 and RIPK3, and MLKL are crucial components in the signaling pathway for necroptosis by the treatment of TNF-α [4,5]. MLKL has been known to serve as an executioner protein via the formation of pores in the plasma membrane during necroptosis, and the pore-forming activity of MLKL occurs upon its oligomerization and translocation of phosphorylated MLKL [14,15]. The phosphorylation of MLKL occurs in a protein complex called a necroptosome, in which RIPK1 and RIPK3 are recruited and activated by phosphorylation [5,13]. Thus, we first investigated whether the NTB451 affected the cellular levels of these components. As shown in Figure 2A, no change in the levels of RIPK1, RIPK3, or MLKL was found in the NTB451-treated cells. Next, we examined whether NTB451 treatment inhibited the modifications of MLKL induced by TNF-α combined with zVAD. In agreement with previous studies [13], the combination of TNF and zVAD led to the phosphorylation and oligomerization of MLKL in L929 cells, and these molecular events on MLKL were prevented by NTB451 treatment in a dose-dependent manner (Figure 2A,B).

As NTB451 treatment prevented the activity in MLKL phosphorylation, we investigated whether NTB451 suppressed TNF-induced necroptosome formation, which is the upstream molecular event of MLKL. To examine the formation of the RIPK1–RIPK3 complex, RIPK3 was immunoprecipitated from cell extracts, and RIPK1 or phosphorylated RIPK1 was probed on a Western blot. As shown in Figure 2C, upon stimulation with TNF-α plus zVAD, the RIPK1–RIPK3 complex was formed, and RIPK1 was phosphorylated. However, treatment with NTB451 or Nec-1 completely blocked both the association between RIPK1–RIPK3 and RIPK1 phosphorylation. According to a previous study, the RIPK1–RIPK3 complex induced by necroptosis had an amyloid structure and was present in detergent-insoluble fractions [12]. Therefore, the effect of NTB451 treatment on the translocation of phospho-RIPK1 and RIPK3 to detergent-insoluble fractions was explored. As expected, NTB451 treatment suppressed the translocation of phospho-RIPK1 and RIPK3 induced by TNF-α and zVAD plus BV6, whereas it did not affect the level of these molecules in detergent-soluble fractions (Figure 2D).

### 2.3. NTB451 Inhibits the Necroptosis by Targeting RIPK1

**Figure 3:**
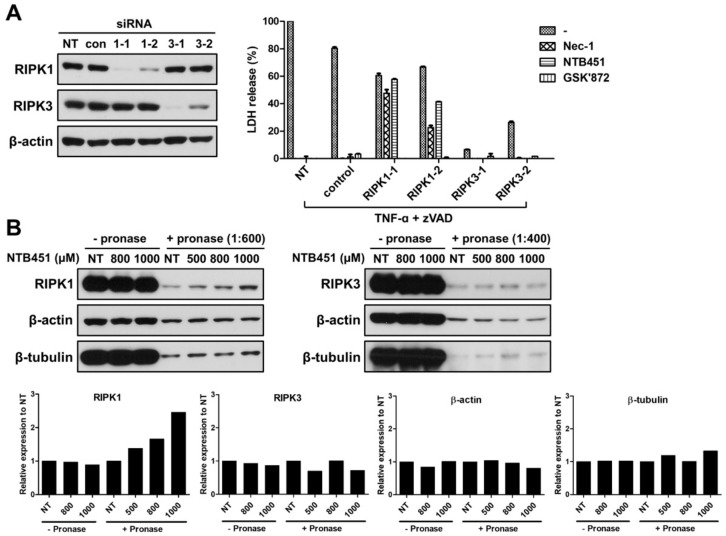
Identification of RIPK1 as a molecular target of NTB451. (**A**) L929 cells were introduced using two different sequences of siRNA and scrambled siRNA control for 48 h. The knockdown efficiency was confirmed by an immunoblot analysis of RIPK1 and RIPK3, with β-actin used as a loading control. The cells were treated with TNF-α (400 units/mL) plus zVAD (20 µM) for 4 h in the presence or absence of Nec-1 (10 µM), NTB451 (20 µM), or GSK’872 (3 µM). The supernatants were then collected, and LDH release was measured. The results are represented as the mean ± standard error of the mean (SEM) of two independent experiments in duplicate wells. (**B**) Drug affinity responsive target stability (DARTS) assay, L929 cells were lysed with TTNE buffer and then incubated with DMSO, the indicated concentration of NTB451 for 30 min. The lysates were digested with pronase (at a 1:600 or 1:400 pronase-to-protein ratio) for 30 min, and immunoblot analysis of RIPK1, RIPK3, β-actin and β-tubulin was performed and the quantitative analysis was done with Image J software. Each bar represents the relative expression level of target protein to untreated (NT) in each group.

NTB451 inhibited the RIPK1RIPK3 interaction triggered by TNF-α; therefore, we further investigated whether RIPK1 or RIPK3 was a direct target of NTB451. It is known that TNF-α-induced necroptosis can occur even in the absence of RIPK1 [8]. To test the inhibitory effect of NTB451 on RIPK1-independent necroptosis, small interfering RNA (siRNA)-mediated RIPK1 knockdown-L929 cells were generated and treated with TNF-α plus zVAD in the presence or absence of NTB451, Nec-1, or GSK’872, an inhibitor of RIPK3. As shown in Figure 3A, TNF-induced cell death occurred in RIPK1 knockdown cells, and the cell death was inhibited by treatment with GSK’872. However, neither NTB451 treatment nor Nec-1 prevented TNF-induced cell death, although they suppressed the cell death of control siRNA-introduced cells. These results indicated that the inhibitory effect of NTB451 on necroptosis may be attributed to its regulation of RIPK1’s function.

Drug affinity responsive target stability (DARTS) is a well-known non-tagged-based technique to confirm the direct molecular target [29]. Therefore, DARTS assay using L929 cells was performed to assess whether NTB451 directly bound to RIPK1. RIPK1 was degraded by a protease pronase, and its proteolysis was decreased by the presence of NTB451 in a dose-dependent manner (left panel of Figure 3B). In contrast, proteolysis of RIPK3 was not affected by NTB451 treatment (right panel of Figure 3B). These results suggested that NTB451 may regulate RIPK1 function via directly targeting RIPK1.

### 2.4. Molecular Docking and Molecular Dynamics (MD) Simulations Demonstrate NTB451 Interacts with RIPK1

**Figure 4:**
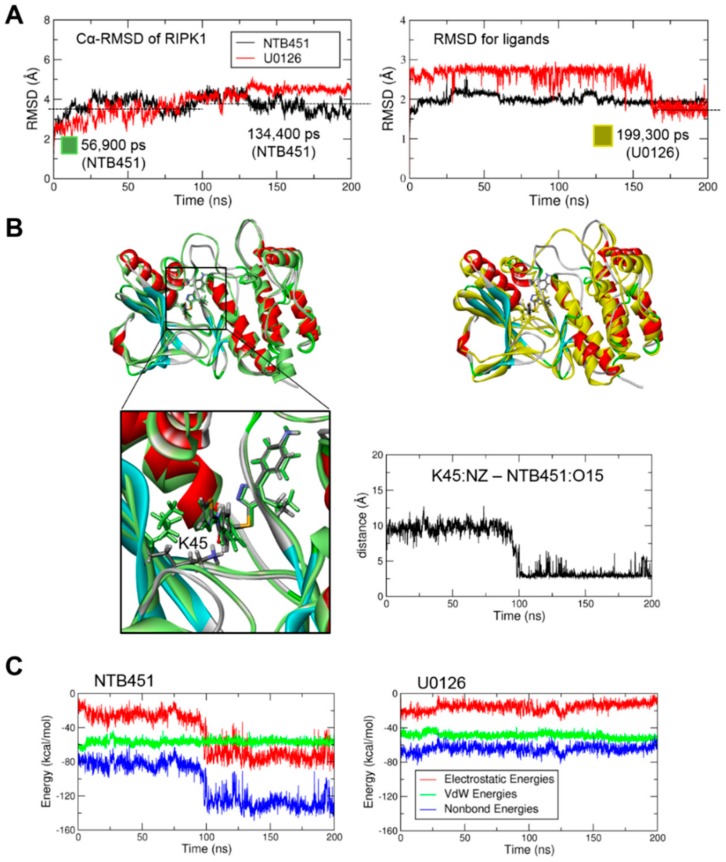
Interaction of NTB451 with RIPK1 investigated by molecular docking and molecular dynamics (MD) simulation studies. (**A**) C-alpha RMSD of RIPK1 (left) and all-atom RMSD of ligands (right) to check the stability of NTB451 (black line) and U0126 (red line) bound systems during the simulation time. (**B**) Snapshots of representative structures taken from the closest snapshots to average during the simulation times. Left upper panel, representative structures of 56,900 pico seconds (ps; green) and 134,400 ps (alpha-helix and beta-sheet colored by blue and red) for the NTB451-bound system to compare before and after 100 ns. Left lower panel, zoomed-in view of the representative structures with NTB451 and key binding residue shown by the stick model. Right upper panel, representative structures of 134,400 ps (NTB451, blue and red) and 199,300 ps (U0126, yellow) to compare active and inactive compound-bound systems. Right lower panel, the distance between the nitrogen atom of the sidechain of K45 and carbonyl oxygen atom of NTB451. (**C**) Time traces of non-bonded energies (blue line), which is the sum of electrostatic (red) and vdW (green) energies for NTB451 (left) and U0126 (right) bound systems.

To further investigate the interaction between NTB451 and RIPK1, we performed molecular docking of NTB451 with RIPK1 and compared the results with those of U0126, which is an inactive compound of RIPK1. Due to the absence of flexibility and structural changes in the binding pocket, the binding strengths of NTB451 and U0126 compounds cannot be distinguished by docking scores obtained from the molecular docking simulation. Hence, to find the difference of their binding intensities after adjusting the flexibility of protein, MD simulations of the docked structures were performed with a 200-ns simulation time. Root-mean-square deviations (RMSDs) of C-alpha atoms of protein and RMSDs of all atoms of ligands were measured to check the system stability of complex structures. During the simulation time, both systems were well equilibrated, and both ligands were adjusted to the binding pocket of RIPK1 (Figure 4A). As shown in the right panel of Figure 4A, the RMSD of inactive compound U0126 (red line) fluctuated, showing less binding affinity. From this RMSD comparison, binding of U0126 was observed to be more unstable than that of NTB451, which was stable and well maintained throughout the simulation time. Next, to quantify these dynamics and to clarify the difference, we calculated the van der Waals (vdW) and electrostatic energies. Then, based on the average structure, representative structures were selected from before and after 100-ns trajectories for further comparative analysis (Figure 4B). Interestingly, we found a dramatic change in the electrostatic energy (left panel of Figure 4C) after the relaxation of NTB451 into the binding pocket of RIPK1. When we compared the representative structures before and after 100 ns, this came from the formation of a strong hydrogen bond interaction between the nitrogen atom of K45 sidechain and carbonyl group oxygen atom of NTB451 (Figure 4B, lower graph). In addition, we found a clear difference between active and inactive compounds through non-bond energy calculations (Figure 4C). From the comparison of interaction energies, the values of non-bond energies of the initial docked structures were similar (approximately −80 kcal/mol), and the value of NTB451 shifted to about −130 kcal/mol after the K45 residue was involved in the ligand binding. However, in the case of the inactive compound, U0126, it remained near −80 kcal/mol throughout the simulation time. These results not only verified the direct interaction of RIPK1, but they also provided structural insight into further lead optimization and structure-based drug design (SBDD) study.

### 2.5. NTB451 Suppresses Toll-Like Receptor 3 (TLR3)- and TLR4-Mediated Necroptotic Cell Death

**Figure 5:**
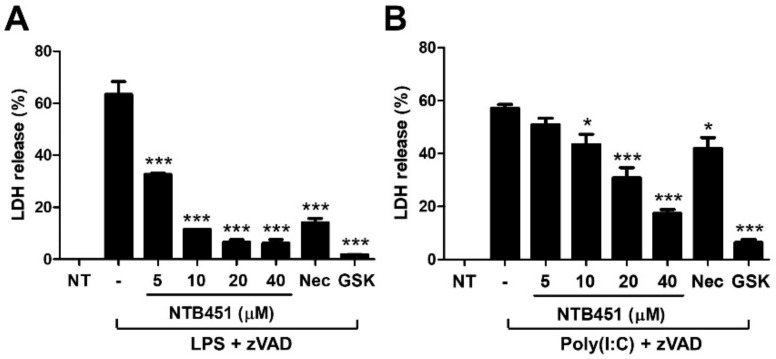
Inhibitory effect of NTB451 on LPS- and poly (I:C)-induced necroptosis. J774A.1 cells were treated with (**A**) LPS (100 ng/mL) plus zVAD (20 µM) or (**B**) poly(I:C) (25 µg/mL) plus zVAD (50 µM) in the presence or absence of the indicated concentrations of NTB451, Nec-1 (10 µM), or GSK’872 (3 µM). The culture supernatants were collected (**A**) 12 h or (**B**) 18 h after treatment, and LDH release was measured. The results are represented as the mean ± standard error of the mean (SEM) of two independent experiments in triplicate wells. * *p* < 0.05, *** *p* < 0.001 compared with LPS + zVAD alone or poly(I:C) + zVAD alone.

It is well documented that necroptosis can occur by triggering of TLR3 or TLR4 in mouse macrophages [30,31]. Here, we investigated whether NTB451 could inhibit necroptosis induced by such TLR agonists. LPS or poly (I:C) combined with zVAD induced cell death of J774A.1 cells, and the death was inhibited by Nec-1 or GSK’872 and NTB451 in a dose-dependent manner (Figure 5A,B). Interestingly, the cell death induced by exposure to poly (I:C) was not suppressed by Nec-1 treatment, whereas it was inhibited by NTB451 and GSK’872 (Figure 5B).

### 2.6. NTB451 Does not Affect TNF-Induced Apoptosis or Nuclear Factor-κB (NF-κB) Signaling

**Figure 6:**
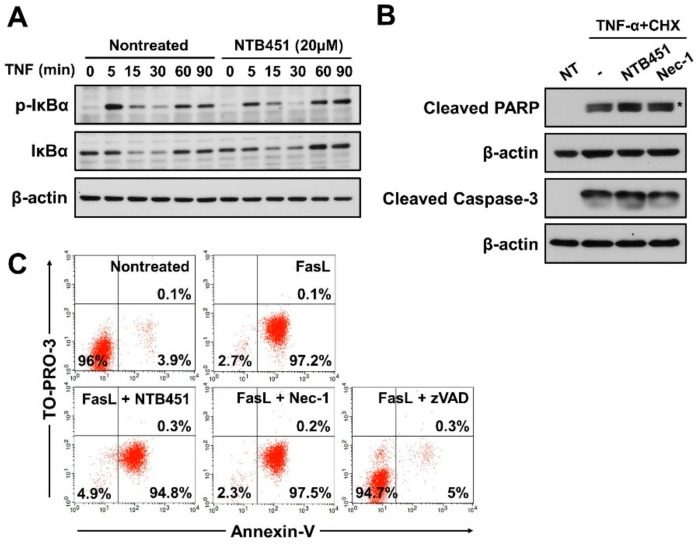
Effect of NTB451 on TNF-α-induced NF-κB activation and on the apoptotic cell death induced by TNF-α and FasL. (**A**) L929 cells were treated with TNF-α (400 units/mL) for the indicated times in the presence or absence of NTB451 (20 µM). Cells were lysed and immunoblotted with the antibodies against phospho-IκB, IκB, and β-actin. (**B**) L929 cells were treated with TNF-α (400 units/mL) plus CHX (10 µg/mL) for 3 h in the presence or absence of NTB451 (20 µM) or Nec-1 (10 µM), and cell apoptosis was analyzed by immunoblotting with anti-cleaved PARP and anti-cleaved caspase-3 in cell lysates. * indicates a nonspecific band. (**C**) Apoptotic cell death in Jurkat cells was induced with 5% FasL for 3 h in the presence or absence of NTB451 (20 µM), Nec-1 (10 µM) or zVAD (20 µM). Then, the cells were stained with PE-conjugated Annexin V and TO-PRO-3 (10 nM), followed by FACS analysis.

Depending on the cellular context, TNF mediates two types of cell death (necroptosis and apoptosis) and NF-κB activation [10,32]. Consistent with the findings of a previous report [33], phosphorylation and degradation of IκB, an inhibitor of NF-κB, was induced 15 min after TNF treatment, and these were not altered by NTB451 treatment at a concentration that effectively inhibited TNF-induced necroptosis (Figure 6A). These results indicated that NTB451 did not influence the TNF-mediated NF-κB signaling pathway.

To explore the effect of NTB451 on the TNF-induced apoptotic cell death, L929 cells were treated with TNF-α plus cycloheximide (CHX) in the presence or absence of NTB451. Upon treatment of the cells with TNF-α/CHX, typical apoptotic markers, such as cleavage of caspase-3 and poly (ADP-ribose) polymerase (PARP), were observed in the cells, and these changes were not suppressed in the presence of NTB451 or Nec-1 (Figure 6B), which suggested that NTB451 did not alter TNF-α-induced apoptotic cell death. To further confirm this notion, the impact of NTB451 on FasL-induced apoptosis in Jurkat cells was examined in a fluorescence-activated cell sorting (FACS)-based assay. As shown in Figure 6C, the FasL treatment of Jurkat cells greatly increased the population of apoptotic cells (Annexin V-positive and TO-PRO-3-negative cells) in 3 h, and the cell death was blocked by zVAD. However, neither NTB451 nor Nec-1 suppressed FasL-induced apoptosis (Figure 6C). Together, these data suggested that NTB451 did not prevent TNF-induced NF-κB activation and TNF- or FasL-mediated apoptotic cell death.

## 3. Discussion

Studies on necroptosis and its inhibitors have increased dramatically in recent years because of its involvement in various physiological and pathological conditions [34,35]. In the present investigation, we identified a chemical, NTB451, which inhibited TNF-induced necroptosis in L929 murine cells (half-maximal inhibitory concentration (IC_50_) = 7.9 µM). Additional analyses revealed that NTB451 activity was not restricted to TNF-induced necroptosis, and it suppressed necroptosis induced by various stimuli, such as poly (I:C) or LPS, in human and murine cells, indicating that NTB451 showed a broad spectrum of activity in the prevention of necroptosis. Further, we characterized the mechanistic basis of NTB451 in the context of current knowledge on cell death by necroptosis.

The data indicated that NTB451 attenuated the key molecular events during necroptosis, including the phosphorylation and oligomerization of MLKL. Earlier studies showed that the necroptosome, which is composed of RIPK1 and RIPK3 kinases, serves as a platform for activation and acts as an essential upstream event for the activation of MLKL [5,13]. In the present study, NTB451 inhibited both the formation of the necroptosome and its translocation to detergent-insoluble fractions, indicating that NTB451 prevented necroptosis via inhibition of necroptosome formation and translocation of phospho-RIPK1 and RIPK3 to detergent-insoluble fractions. In addition, experiments using RIPK1 knockdown cells and the DARTS assay revealed that NTB451 directly targeted RIPK1. Based on these data, NTB451 seems to function in a similar manner to that of Nec-1, which inhibited necroptosis by blocking the function of the RIPK1 kinase. Further molecular dynamic simulation study revealed that NTB451 indeed interacted with K45 of RIPK1, which mediates kinase activity. NTB451 has a structural similarity at their benzyl-triazol moiety with some known RIPK1 inhibitors, Nec-1, GSK 2982772 [23,36]. However, the functional relationship of such moiety needs to be elaborately studied.

Previous reports revealed that TNF was able to induce both a prosurvival pathway and two different types of cell death pathways by assembling different signaling complexes, namely complexes I, IIa, and IIb, and this depended on the cellular context [8,9]. Therefore, it is possible that NTB451 can inhibit multiple pathways induced by TNF. To clarify this issue, we examined the effect of NTB451 on both TNF-induced apoptosis and NF-κB activation. Unlike the inhibitory effect of NTB451 on TNF-induced necroptosis, NTB451 had no effect on either NF-κB activation or apoptotic cell death induced by the same ligand; this indicates that NTB451 has a high specificity for necroptosis inhibition. However, further studies are essential for understanding the intrinsic mechanism involved in the binding of NTB451 to RIPK1 and how this binding is associated with RIPK1 kinase activity to develop NTB451 as a drug-like necroptosis inhibitor.

## 4. Materials and Methods

### 4.1. Reagents

The Chemical Library was obtained from Korea Chemical Bank. 4-({[5-(4-aminophenyl)-4-ethyl-4H-1,2,4-triazol-3-yl]sulfanyl}methyl)-N-(1,3-thiazol-2-yl)-benzamide (NTB451, cat. no. STK205750) was purchased from Vitas-M Laboratory (Champaign, IL, USA). Recombinant murine or human TNF-α was obtained from Peprotech (Rocky Hill, NJ, USA). LPS (*Escherichia coli* 011:B4) and CHX were from Sigma-Aldrich (St. Louis, MO, USA). Poly (I:C) was from InvivoGen (San Diego, CA, USA). z-Val-Ala-Asp-(OMe)-fluoro-methylketone(zVAD-FMK) and N,N′-(hexane-1,6-diyl)bis(1-{(2S)-2-cyclohexyl-2-[(N-methyl-L-alanyl)amino]acetyl}-L-prolyl-beta-phenyl-L-phenylalaninamide) (BV6) were from ApexBio (Boston, MA, USA). Nec-1 and GSK’872, known necroptosis inhibitors, were obtained from Santa Cruz Biotechnology (Dallas, TX, USA) and BioVision (Milpitas, CA, USA), respectively. A 293T cell culture supernatant containing FasL was prepared as previously described [37].

### 4.2. Cell Culture

The human embryonic kidney cell line, 293T (ATCC^®^ CRL-3216™), and murine fibroblast cell line, L929 (ATCC^®^ CCL-1™), were maintained in Dulbecco’s Modified Eagle’s Medium (DMEM) supplemented with 10% fetal bovine serum (FBS) and 1% antibiotics (100 U/mL of penicillin, 100 µg/mL of streptomycin) at 37 °C in 5% CO_2_ and immortalized murine embryonic fibroblast (MEF) prepared from 12.5 dpc embryo, as described elsewhere [38]. Human colorectal adenocarcinoma cell line HT-29 cells (ATCC^®^ HTB-38™) were maintained in McCoy’s 5A Modified Medium with 2 mM L-glutamine, 10% FBS and 1% antibiotics. A murine macrophage cell line, J774A.1 (ATCC^®^ TIB-67™), and human T lymphoblastoid cell line, Jurkat (ATCC^®^ TIB-152™), were cultured in RPMI 1640 medium supplemented with 10% FBS and 1% antibiotics.

### 4.3. Antibodies

The following antibodies were used in this study: anti-RIPK1 (BD Biosciences, San Jose, CA, USA), anti-phospho-RIPK1 (Ser166; Cell Signaling Technology, Danvers, MA, USA), anti-phospho-hRIPK1 (Ser166 [39]; Cell Signaling Technology), anti-RIPK3 (Novus Biologicals, Littleton, CO, USA), anti-phospho-hRIPK3 (Ser227) (Cell Signaling Technology), anti-MLKL (Abgent, San Diego, CA, USA), anti-phospho-MLKL (Abcam, Cambridge, UK), anti-β-actin (Sigma-Aldrich, USA), anti-β-tubulin (Cell Signaling Technology), anti-cleaved caspase-3 (Cell Signaling Technology), anti-cleaved PARP (Cell Signaling Technology), anti-phospho-IκBα (Cell Signaling Technology), and anti-IκBα (Santa Cruz Biotechnology).

### 4.4. Screening of the Chemical Library

A representative chemical library (8363 compounds) was provided from Korean chemical bank and the library composed of structurally diversified compounds donated from various organizations in the republic of Korea. Screening was carried out through the inhibitory effect of chemicals on TNF-induced necroptotic death of L929 cells. L929 cells (1.5 × 10^4^) on 96 well plates were treated with TNF-α (400 units/mL) plus zVAD-FMK (20 µM) with or without 50 µM of chemicals. Nec-1 (10 µM) was used as a positive control in each experiment. Five hours after incubation, the viable cells were measured using a crystal violet assay [39]. Briefly, the cells were washed twice with phosphate-buffered saline (PBS) and stained in crystal violet solution consisting of 0.5% crystal violet (Sigma-Aldrich, USA) and 20% methanol in distilled water for 20 min at room temperature. The cells were then washed with tap water and dried completely. The stained cells were treated with methanol for dissolving, and the dye was dissolved in a 200-µL volume of methanol. The absorbance (wavelength: 570 nm) was measured using a Multiskan GO Microplate Spectrophotometer (Thermo Fisher Scientific, Waltham, MA, USA).

### 4.5. Lactate Dehydrogenase (LDH) Assay and Half-Maximal Inhibitory Concentration (IC_50_) Determination

Cell cytotoxicity was determined by LDH release in culture supernatant according to the manufacturer’s instructions (DoGenBio, Seoul, Korea). Briefly, the cells were treated with the indicated concentration of inhibitors and TNF-α (400 units/mL), LPS (100 ng/mL), or poly (I:C) (25 µg/mL) plus zVAD-FMK (20 µM) for the indicated times. The supernatants were collected after centrifugation at 300× *g* for 5 min. IC_50_ value (the chemical concentration that inhibits 50% cell death compared with the vehicle treated controls) was calculated by GraphPad Prism 5.0 software (log [inhibitor] vs. normalized response).

### 4.6. Immunoblotting

The cells were lysed with RIPA buffer (iNtRON, Sungnam, Korea) added to a protease inhibitor cocktail, and the protein concentration was determined using a Pierce BCA Assay Kit (Thermo Fisher Scientific). The protein lysates were processed by sodium dodecyl sulfate (SDS)–polyacrylamide gel electrophoresis to separate the proteins and electrically transferred to a nitrocellulose membrane. The membranes were then blocked with 5% skim milk in PBST for 1 h at room temperature and blotted with primary antibodies overnight at 4 °C. After washing the membrane with PBST, the membranes were incubated with horseradish peroxidase–conjugated secondary antibody for 1 h. An enhanced chemiluminescence kit (Thermo Fisher Scientific or Merck Millipore, Burlington, MA, USA) was used for visualization of secondary antibodies, using either X-ray film or a Davinch–Chemi imaging system.

For detecting MLKL oligomerization by immunoblotting, the cells were lysed with TTNE buffer (1% Triton X-100, 50 mM Tris, 150 mM NaCl, and 2 mM EDTA) added a protease inhibitor cocktail. The protein concentration was then measured using a BCA assay. Subsequently, the lysates were boiled with a sample buffer without β-mercaptoethanol (non-reducing condition) and analyzed by immunoblotting. For the quantitative analysis of the amount of protein, the integrated optical density of the protein bands was calculated by Image J Software (1.52e, National Institutes of Health, Bethesda, MD, USA).

### 4.7. Co-Immunoprecipitation Assay

TTNE buffer–added protease inhibitor cocktail was used for lysis of L929 cells. After determining the protein concentration through a BCA assay, an equal amount of each sample was incubated with anti-RIPK3 antibody (2 µg/mL) overnight at 4 °C. The samples were then incubated with protein G agarose beads (Millipore, USA) for 4 h at 4 °C. The complexes were eluted by boiling with 2× sample buffer after washing five times with TTNE buffer and analyzed by a Western blot with anti-ph-RIPK1 antibody.

### 4.8. Triton X-100 Fractionation

To obtain detergent-soluble and insoluble fractions, HT-29 cells were lysed with TTNE buffer and incubated for 20 min on ice, followed by centrifugation at 3500× *g* for 10 min. The supernatant (soluble fraction) was collected and stored at −20°C. The pellets (insoluble fraction) were washed with TTNE buffer and lysed by boiling and overnight incubation in 1% SDS lysis buffer (1% SDS, 20 mM Tris-HCl, 150 M NaCl). The lysates were then harvested by centrifugation at 16,200× *g* for 15 min after sonication (YUJINSM, Seoul, Korea) at an amplitude of 20% for 10 s. Subsequently, the supernatants were boiled with sample buffer and analyzed by a Western blot with anti-RIPK1 and anti-RIPK3.

### 4.9. siRNA-Mediated Gene Knockdown

The sequences of synthetic siRNAs (Bioneer, Daejeon, Korea) for RIPK1 and RIPK3 were as follows: RIPK1-1: 5′-CCA CUA GUC UGA CUG AUG A-3′, 5′-UCA UCA GUC AGA CUA GUG G-3′, RIPK1-2: 5′-GAG GAU AUU CUC AGG CUU CAG GUC CUU-3′, 5′-AAG GAC CUG AAG CCU GAG AAU AUC CUC-3′, RIPK3-1: 5′-CCC GAC GAU GUC UUC UGU CAA-3′, 5′-UUG ACA GAA GAC AUC GUC GGG-3′, RIPK3-2: 5′-AAG AUU AAC CAU AGC CUU CAC CUC CCA-3′, 5′-UGG GAG GUG AAG GCU AUG GUU AAU CUU-3′. Lipofectamine 3000 (Thermo Fisher Scientific) was used for transfection of siRNA according to the manufacturer’s instructions. L929 cells were transfected with 50 nM siRNA for at least 48 h and used for further experiments.

### 4.10. DARTS Assay

DARTS assay was performed according to the protocol described in a previous study [40]. L929 cells were lysed with TTNE buffer and then incubated with dimethyl sulfoxide (DMSO), NTB451 for 30 min. After incubation, the lysates (3 µg/µL) were digested with pronase (1:600 or 1:400 pronase-to-protein ratio) for 30 min. Digestion of lysates was stopped by incubation with 20× protease inhibitor cocktail for 10 min and then boiling with a sample buffer, followed by immunoblot analysis.

### 4.11. Molecular Docking Simulations

Molecular docking simulations were performed using CDOCKER [41] implemented in Discovery Studio (DS) 2018 software [42]. We used this program to generate the initial docking poses of NTB451 and U0126, which were needed for the following molecular dynamics (MD) simulation study. The crystal structure of RIP1 kinase, bound to inhibitor (PDB ID: 6C3E) [36] with a resolution of 2.6 Å, was used for the validation of the docking simulation. The active site of RIPK1 was defined from the bound inhibitor, 2-benzyl-5-nitro-1H-benzimidazole, within a radius of 10 Å. For the flexibility of the ligand, 100 conformations were generated through random rotations and high temperature (1000 K) MD simulation (1000 steps). The conformations were fitted into the defined active site by simulated annealing refinement.

### 4.12. Molecular Dynamics (MD) Simulations

Although the initial docking poses of NTB451 and U0126 were generated by the validated docking method, it could not make a difference in the docking score between NTB451 and U0126 due to a lack of protein flexibility. To overcome this obstacle of the docking simulation, we performed the all-atom MD simulations of the docked structures by GROMACS2016 with a CHARMM36 force field. The CHARMM-GUI [43,44] was used for the generation of input for the simulations. The topologies and parameters of NTB451 and U0126 were generated by the CHARMM General Force Field (CGenFF) program [45]. The cubic water boxes with a 10 Å thicknesses solvated by the TIP3P water model were constructed for the systems. The steepest descent energy minimization was conducted to remove steric clashes or possible bad contacts until a tolerance of 1000 kJ/mol. Consequently, the minimized structures with restrained heavy atoms were subjected to the NVT equilibration process during the 25 ps with a 1-fs time step at 303.15 K. The LINCS algorithm [46] was used to constrain the bonds between the heavy atoms and corresponding hydrogen atoms by their equilibrium bond lengths. Finally, the production runs for 200 ns were performed by NPT dynamics with a constant temperature and pressure (303.15 K and 1 bar) achieved with a Nosé–Hoover thermostat [47,48] and Parrinello-Rahman [49] barostat. A time step of 2 fs was applied for the production runs and the trajectory was recorded every ps. The cut-off values of the short-range interactions and vdW interactions were set to 12 Å. The particle-mesh Ewald method [50] was used for the long-range electrostatic interactions. To analyze the protein–ligand interactions, representative structures were selected as the closest frame to average during the first 100 ns and last 100 ns for the NTB451-bound system. For U0126, due to the highly unstable fluctuation of the ligand, we selected the representative structure during the last 35 ns.

### 4.13. FACS Analysis

Jurkat cells were treated with 5% FasL and 20 µM NTB451, 10 µM Nec-1, or 20 µM zVAD. After incubation for 3 h, the cells were centrifuged at 250× *g* for 5 min with same volume of 1× annexin-V binding buffer (10 mM HEPES buffer [pH 7.4], 140 mM NaCl, and 2.5 mM CaCl_2_). The cell pellets were suspended with annexin-V solution (5 µL of annexin-V-PE (BD Biosciences) per sample in 1× annexin-V binding buffer) and incubated at room temperature for 15 min in the dark. The annexin-V-stained cells were treated with 1 nM TO-PRO3 iodide (Thermo Fisher Scientific) and analyzed using an FACS machine (BD Biosciences).

### 4.14. Statistical Analysis

The results are expressed as the mean ± standard error of the mean (SEM). The statistical analysis was assessed either by Student’s *t*-test or one-way analysis of variance (ANOVA) using GraphPad Prism 5.0 (La Jolla, CA, USA). Values of *p* < 0.05 were considered statistically significant.

## 5. Conclusions

In summary, considering the overall data obtained, our study indicated that the new compound NTB451 has immense potential in selectively inhibiting the necroptosis pathway. Moreover, it may be developed as a therapeutic agent in the treatment of a variety of diseases that have a necrosis phenotype involving necroptotic cell death.
